# Enhanced Diffusion of Single, Lipid-Tethered Enzymes

**DOI:** 10.1021/acs.nanolett.5c05619

**Published:** 2026-02-14

**Authors:** Ashley Scott, Mengqi Xu, Ian Murphy, David S.-J. Jang, Zainab Marwa Rana, Anthony Estrada, Wylie W. Ahmed, W. Benjamin Rogers, Jennifer L. Ross

**Affiliations:** ‡ Physics Department, 2029 Syracuse University, Syracuse, New York 13244 United States; § Martin A. Fisher School of Physics, 8244 Brandeis University, Waltham, Massachusetts 02453 United States; ⊥ Laboratoire de Physique Théorique and Centre de Biologie Intégrative, Université de Toulouse, CNRS, Toulouse 31078, France; ¶ Department of Physics, California State University, Fullerton, California 92831, United States

**Keywords:** particle tracking, total internal reflection fluorescence microscopy, nanoscale active matter, nonequilibrium, DNA linker, fluorescence recovery after photobleaching, mobility

## Abstract

Recent experimental evidence has shown that enzymes that catalyze exergonic reactions are able to diffuse faster during catalysis, a process called “enhanced diffusion”. If true, enzyme propulsion could enable the engineering of designed active materials at the nanoscale. However, further experimental validation is needed under well-controlled conditions. We use single-molecule tracking of enzymes tethered to fluid lipid bilayers, which serve to constrain motion to two dimensions, lower baseline diffusion for improved sensitivity, and accommodate multiple tethering strategies. We find that active urease diffuses approximately 40% faster in the presence of substrate (urea) than in its absence or when inhibited, independent of the tethering scheme. The degree of enhancement scales with the substrate concentration, consistent with prior studies. Finally, we find that assembling multiple enzymes into larger complexes results in even greater diffusion enhancement. This work indicates that enzymes could serve as a platform to create and study active particles at the nanoscale.

## Graphical abstract

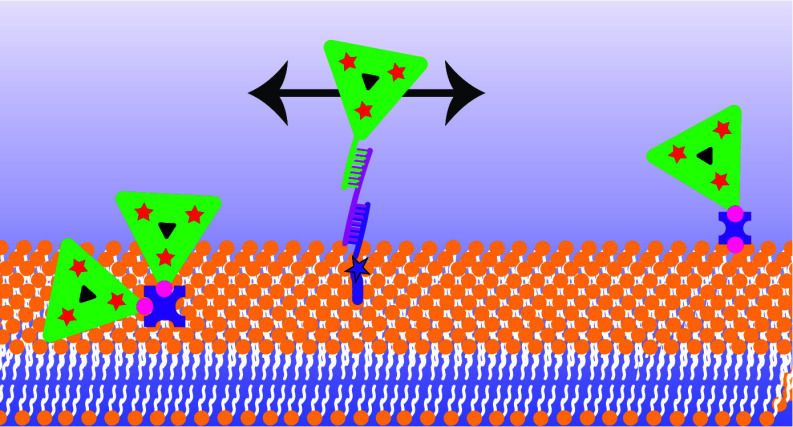

Enzymes, proteins that catalyze chemical reactions, have been likened to nanoscale, active colloidal particles that bind reactants and release products to self-propel.^1–6^ Active colloids are typically micrometer-scale synthetic particles asymmetrically coated with enzymes or other catalysts to produce solute gradients that can push or pull the particles.^7–9^ The motion of active colloids can be characterized by the Péclet number, *Pe*, a dimensionless ratio of the advective transport rate over the diffusive transport rate. Large colloids have *Pe* > 1 because their rotational diffusion is slow, resulting in long persistence times and predominantly ballistic transport. In contrast, the small size of enzymes leads to high translational and rotational diffusion rates, while the relatively low catalytic turnover results in a low advection rate. Thus, for a single enzyme, *Pe* ≪ 1, and any activity-induced motion manifests as ’enhanced diffusion’, or an increase in apparent diffusivity upon increasing substrate concentration.^10^ Over the past decade, several studies have demonstrated that exergonic enzymes (Δ*G* < 0) with high turnover rates exhibit enhanced diffusion,^2,4,5,11–16^ while other studies have shown no enhancement.^13,17–20^

If enzymes could function as propulsive subunits, they could serve as an engineering platform to create novel active particles at the nanoscale. Understanding the fundamental engineering principles would allow us to make active, hierarchically assembled materials with advanced properties, such as the abilities to sense and respond to the environment. Moreover, studying nanoscale active systems is essential for uncovering the physical mechanisms underlying biological processes in living cells. Establishing the physical limits and mechanisms of enzyme-driven motion is key to both advancing basic science and enabling future applications

Here, we perform a series of experiments that quantify the mobility of individual urease enzymes tethered to fluid lipid bilayers using optical microscopy and single-particle tracking. Membrane tethering has several advantages over prior single-molecule methods. First, the lipid bilayer imposes drag on tethered objects, slowing their diffusion within the two-dimensional fluid. This reduced diffusion rate makes it easier to characterize enzyme motion using standard imaging methods. Second, constraining the enzyme’s motion to two dimensions prevents it from leaving the imaging plane, allowing for longer continuous tracking of individual molecules (Figure 1A,B). Third, membranes support multiple well-established biomolecular tethering strategies, enabling cross-validation with different conjugation methods to rule out artifacts from any single approach.

**Figure 1:**
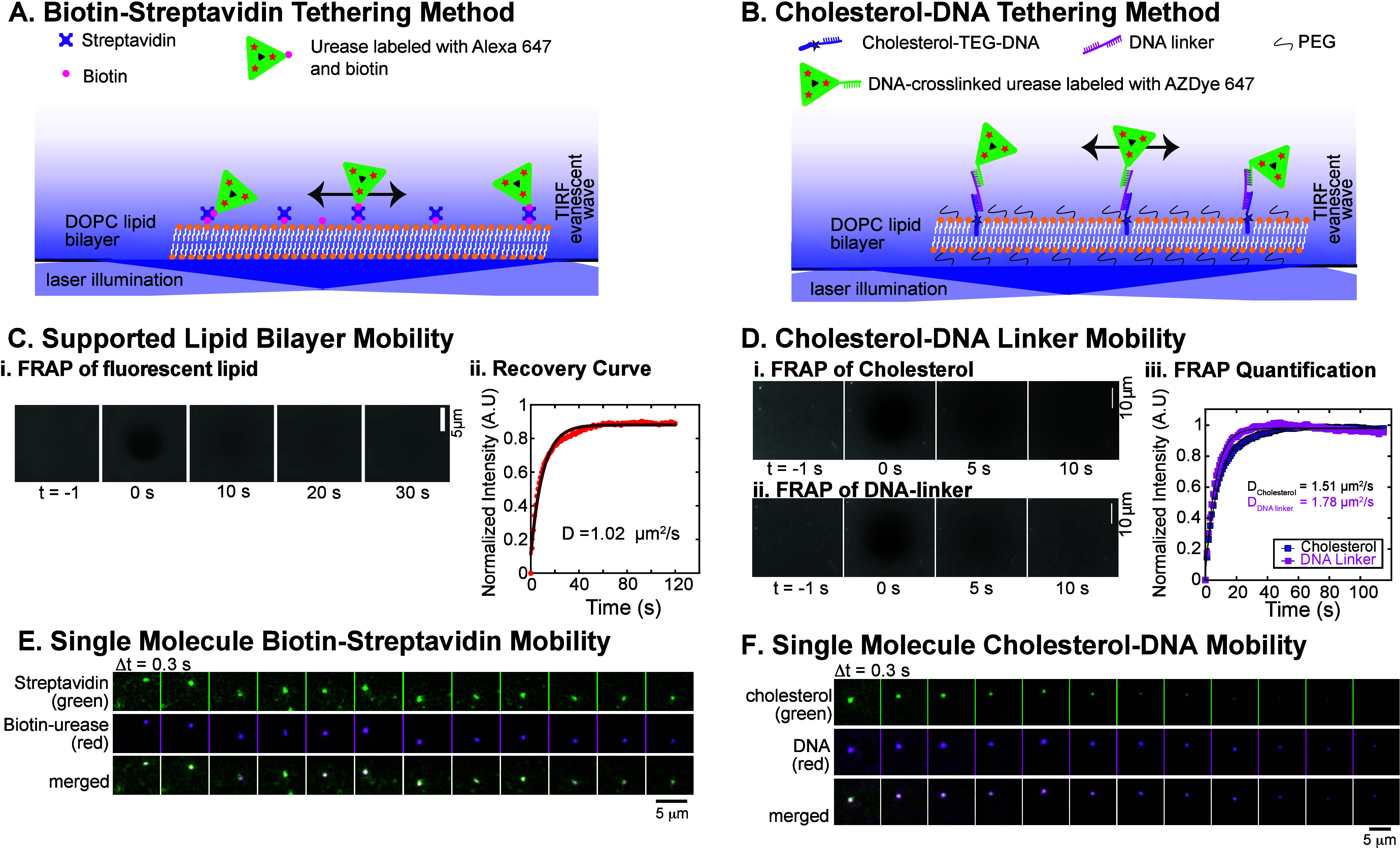
Tethering schemes to attach enzymes to fluid lipid bilayers. (A) Biotin–streptavidin tethering scheme showing urease enzymes (green triangles) labeled with Alexa-647 (red stars) and biotin (magenta circles). Biotinylated PE lipids mixed with DOPC lipids (orange and white) to create a fluid lipid bilayer that can bind streptavidin (purple crosses). Single enzymes are imaged with total internal reflection fluorescence microscopy. (B) DNA–cholesterol tethering scheme showing DNA-cross-linked urease enzymes (green) labeled with AZDye-647 (red stars). The ssDNA cross-linked to the urease enzymes bind to the complementary 15 nucleotide domain of the DNA linker (magenta). Cholesterol–TEG–DNA (purple) binds to the complementary 17 nucleotide domain of the DNA linker. PEG2000 PE lipids (black) are mixed with DOPC lipids (orange and white) to create a fluid lipid bilayer that can bind the hybridized DNA linkers. (C) FRAP of Texas-Red-labeled lipid bilayers. (i) Time series of photobleaching and recovery of the lipid bilayer. (ii) Fluorescence intensity over time fit to exponential rise. Fit parameters are given in Supp. Table 1 FRAP of hybridized cholesterol–DNA linkers labeled with 6-FAM and Alexa-647. Time series of photobleaching and recovery of (i) cholesterol–TEG–DNA (6-FAM) and (ii) DNA linker (Alexa-647) on lipid. (iii) Fluorescence intensity over time fit to exponential rise with fit parameters given in Supp. Table 1. (E) Time series of single-molecule biotin–streptavidin complexes showing (i) streptavidin, (ii) biotin–urease, and (iii) merging of the images. (F) Time series of single-molecule cholesterol–DNA complexes showing (i) cholesterol, (ii) DNA, and (iii) merging of the images. Scale bars are as reported for each set of images.

We use two conjugation strategies to tether urease enzymes to a supported phospholipid bilayer. The first method uses streptavidin to cross-link biotinylated enzymes to biotinylated lipids (Figure 1A). The second method uses hybridization of cholesterol-modified single-stranded DNA to an enzyme conjugated to the complementary sequence of single-stranded DNA (Figure 1B).^21,22^ Daily, we verify the fluidity and homogeneity of the supported lipid bilayer using fluorescence recovery after photobleaching (FRAP). Figure 1C shows the representative timeseries and quantitative FRAP analysis of fluorescent lipids. We find that the lipid bilayer exhibits efficient recovery, with diffusion coefficients within the expected range, *D* = 1–2 μm^2^/s (Figure 1C).^23–25^ FRAP experiments performed with fluorescently labeled cross-linkers show that they are also mobile (Figure 1D), with a diffusion coefficient similar to the lipids (Figure 1C,D and Supp. Figure 1). We further quantify the diffusion of single cross-linked complexes using two-color simultaneous single-molecule imaging and tracking. We show that the fluorescent streptavidin–biotin–urease (Figure 1E) and cholesterol–DNA (Figure 1F) conjugates diffuse together, confirming that the enzyme-cross-linker complexes move cohesively within the fluid bilayer.

To deduce the enzyme diffusion coefficients, we image individual enzymes using total internal reflection fluorescence microscopy (TIRF) and track their motion with standard image-analysis routines (Figure 2A; see Supp. Methods and Supp. Figure 2).^4^ The resulting trajectories are then used to calculate the mean squared displacement (MSD) as a function of the lag time, τ, and fit to the linear equation ⟨*r* ^2^⟩ = 4*D*τ^α^, where *D* is the diffusion coefficient, α is the anomalous diffusion exponent, and the prefactor of 4 accounts for two-dimensional motion [Figure 2A­(ii)]. This process is repeated for approximately 100 particles in each experimental condition. We find that the anomalous diffusion exponent is equivalent to 1, within 4% uncertainty, for all fits, indicating that the measured single-molecule diffusion is purely Brownian and neither superdiffusive nor subdiffusive. This result is consistent with previous findings.^4^

**Figure 2:**
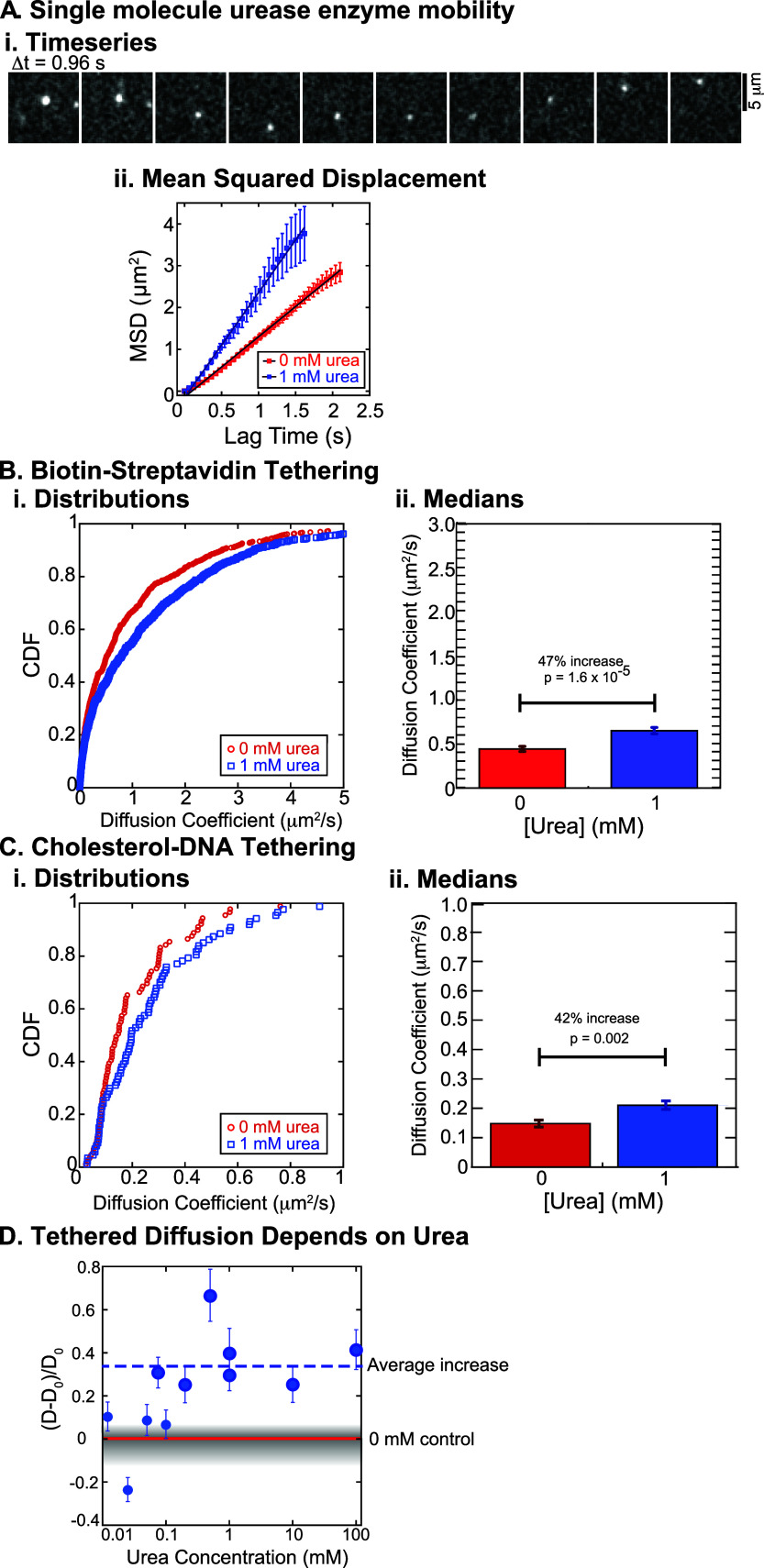
Diffusion measurements of urease. (A) (i) Quantification of the time series of single urease diffusion. (ii) Mean-squared displacement without urea (red squares) and with 1 mM urea (blue squares). Error bars represent SEM. Data are fit to a line to determine the diffusion coefficient, and fit parameters are given in Supp. Table 2. (B) (i) Biotin–streptavidin-tethered enzymes data plotted as a CDF of the diffusion coefficients comparing 0 mM urea (red open circles) and 1 mM urea (blue open squares). (ii) Bar chart of median values comparing 0 mM urea (red bar) and 1 mM urea (blue bar). Error bars represent SEM. (C) (i) Cholesterol–DNA-tethered enzymes data plotted as CDFs for 0 urea (red open circles) compared to 1 mM urea (blue open squares). (ii) Bar chart of median values comparing 0 mM urea (red bar) and 1 mM urea (blue bar). Error bars represent SEM. (D) Diffusion coefficients normalized with respect to same day control experiments in the absence of urea (*D*_0_) plotted as a function of the urea concentration. Points that are larger and with darker exterior circle are statistically significant compared to the same-day control with *p* ≤ 0.01. The horizontal blue dashed line denotes the average increase for all larger points.

We determine the cumulative distribution functions (CDFs) of the diffusion coefficients for each condition and compare them across various urea concentrations (Figure 2B,C). We use the median value (defined as the location where the CDF equals 0.5) of each CDF to represent the apparent diffusion coefficient for each experimental condition (see Supp. Methods for details).

Using either tethering scheme, we find that lipid-tethered enzymes exhibit enhanced diffusion upon increasing substrate concentration. For the biotin–streptavidin tethering condition, the addition of 1 mM urea shifts the distribution of diffusion coefficients toward values approximately 47% higher than those observed without urea (Figure 2B). We compare the distributions using the Kolmogorov–Smirnov statistical test (KS Test) and find that the change is statistically significant [*p* = 1.6 × 10^–5^; Figure 2B­(ii)]. We observe a similar, statistically significant increase in the diffusion coefficient of 42% using the cholesterol–DNA system (*p* = 0.002, KS Test; Figure 2C). Importantly, FRAP measurements on bilayers with and without 1 mM urea show no difference in lipid recovery, confirming that the lipid bilayer itself is not affected by the presence of urea (Supp. Figure 4). The consistency between these two tethering schemes supports the robustness of the observed enhancement. Furthermore, the magnitudes of the enhanced diffusion are in line with prior results of urease-catalysis-induced diffusion enhancement in free solution measured by fluorescence correlation spectroscopy.^2,12–14^

Notably, the distributions of diffusion coefficients determined from single-particle tracking are broad, often spanning over an order of magnitude, as previously observed.^26–29^ Although limited trajectory lengths due to photobleaching can contribute to this broadness,^26^ we suspect the dominant factor here is likely heterogeneity of the lipid bilayer in the local environment of the molecules being tracked. Therefore, to detect shifts in the distributions reliably, it is essential to compare experiments conducted under identical lipid and molecular compositions, include proper controls, and analyze a substantial number of molecules.^27^ We also find that the basal diffusion coefficients for urease tethered via cholesterol–DNA are consistently slower than those obtained using biotin–streptavidin linkage (compare parts B­(ii) and C­(ii) of Figure 2). This result is not surprising considering the cholesterol can cause the lipids in the bilayer to order and thereby reduce their mobility at high cholesterol concentrations.^30,31^ When we quantify both the diffusion and fluorescence intensity of the cholesterol anchors, we also find that the slower cholesterol complexes tend to be brighter (Supp. Figure 5), suggesting that cholesterol may also form small complexes within the bilayer.

We seek to identify at what concentration of urea the diffusion is altered and if the effect saturates. We measure single-molecule diffusion across 4 orders of magnitude of urea concentrations (0.01–100 mM). To enable direct comparison across different conditions, we first measure the diffusion coefficient (*D*_0_) in the urea-free control and then measure *D* in the presence of urea within the same sample chamber, and quantify the relative increase in *D* for each concentration. By sequentially increasing urea concentration within the same sample chamber, we ensure identical lipid composition and protein preparation across all measurements, thereby allowing direct and reliable comparisons between conditions, as discussed above. We plot the change in diffusion coefficient of urease as a function of urea concentration (Figure 2D). We find that the diffusion coefficient at the lowest urea concentrations (0.0125–0.05 mM) is slightly higher but statistically indistinguishable from the urea-free control measured on the same day (Figure 2D, small symbols; see Supp. Table 5 for statistics). At 0.075 mM, the diffusion coefficient is 31% higher than the control. Between 0.075 and 0.5 mM, the diffusion coefficient increases and then plateaus above 1 mM (Figure 2D, larger symbols). In this range of urea concentrations, the diffusion coefficient increased by an average of 34 ± 6% (with a range of 25–66%, error indicated SEM, average denoted with the dashed line in Figure 2D). Using the KS Test, we show that each of these increases are statistically significant compared to same day, urea-free controls (see Supp. Table 5). The average increase we observe is similar to the enhancement observed in several prior studies of freely diffusing urease quantified by other methods.^2,12–14^ The clear urea-concentration dependence of our findings suggests that the enzyme binding to substrate or enzymatic activity is associated with enhanced diffusion.

To test if the enhanced diffusion is due to the enzyme activity, we measure the mobility of urease inhibited with a small molecule called catechol (see Supp. Methods).^32,33^ We verify urease inhibition by monitoring absorbance at 560 nm in a plate-reading spectrophotometer with 70 μM phenol red, a pH indicator, which is yellow at and below pH 6.8 and turns red as pH increases up to pH 8.2. Because urease produces basic reaction products, phenol red provides a sensitive readout of enzyme activity.^4,34^ In our assay, the untreated enzyme shows increased absorbance at 560 nm, while the inhibited enzyme shows no change, confirming successful inhibition (Figure 3A).

**Figure 3:**
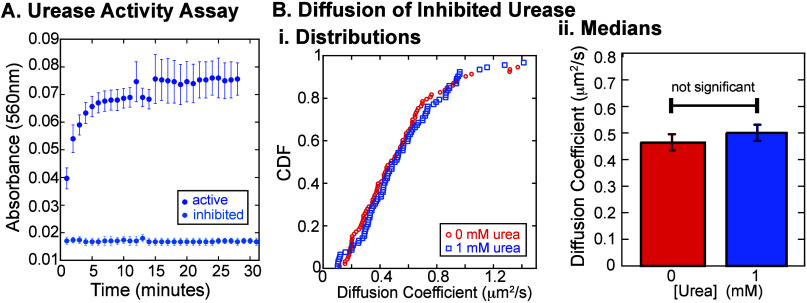
Inhibition of urease negates enhanced diffusion of tethered urease. (A) Spectroscopic urease activity assay comparing active urease enzymes (blue circles) with inhibited urease enzymes (blue-gray circles) in the presence of 5 mM urea for both. (B) Diffusion of single enzymes. (i) Cumulative distributions of diffusion coefficients of inhibited single-molecule urease enzymes in the presence of 1 mM urea (blue squares) and uninhibited urease enzymes in the absence of urea (red squares). (ii) Bar charts showing no difference between inhibited urease with 0 urea (red bar) and 1 mM urea (blue bar). Error bars represent SEM.

Using inhibited enzymes, we perform the same type of single-molecule measurements of diffusion in the same chamber with both 0 and 1 mM urea. We observe no significant change in the diffusion coefficient of inhibited urease in the presence of urea compared to the control [Figure 3B­(i,ii), *p* = 0.62]. This result supports the conclusion that enhanced diffusion of urease is driven by its catalytic activity, and adds further evidence that the urea is unlikely to change the fluidity of the lipid bilayer itself.

Our experiments imply that catalytic activity correlates with enhanced diffusion. Thus, we hypothesize that increasing the number of active sites per complex could further amplify this enhancement. To test this idea, we leverage the multivalency of streptavidin to create larger complexes of biotinylated urease by mixing streptavidin with biotinylated enzymes. Each streptavidin molecule possesses four biotin-binding sites, allowing it to bind 1–3 enzymes while retaining an available site for attachment to the biotinylated lipid bilayer, enabling single-molecule tracking (Figure 4A). Further, if complexes are made with multiple streptavidins, even more enzymes can be incorporated into the multienzyme complexes, providing more available catalytic sites.

**Figure 4:**
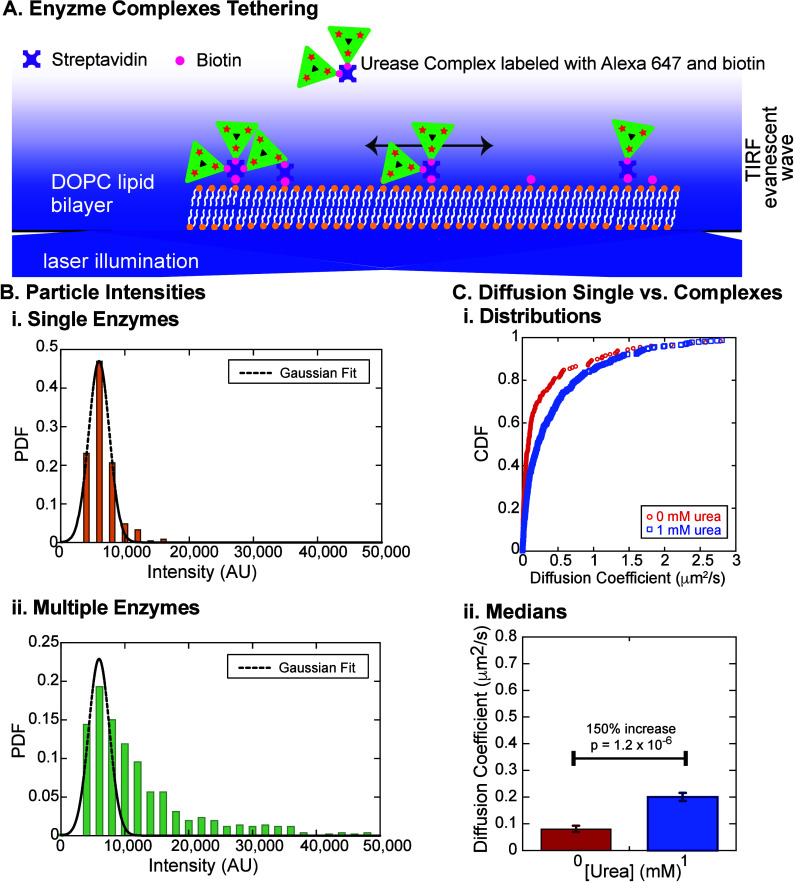
Diffusion of multimeric enzyme complexes tethered to lipid bilayer via biotin and streptavidin. (A) Cartoon of multimeric enzyme complexes tethered to lipid bilayer via biotin and streptavidin. (B) Probability distributions of particle intensities for (i) single urease molecules (orange bars, *N* = 178) fit to a Gaussian distribution and (ii) multimeric enzyme complexes (green bars, *N* = 484) with the same parameters as the fit to the single particles except the amplitude is allowed to change. All fit parameters are given in Supp. Tables 7 and 8. (C) Cumulative distributions of diffusion coefficients for multimeric enzyme complexes with 0 mM urea (red open circles) and 1 mM urea (blue open squares). (ii) Bar plots comparing multimeric enzyme complexes with 0 urea (red bar) and 1 mM urea (blue bar). Error bars represent SEM.

To validate this multiple-enzyme-complex assembly strategy, we estimate the number of enzyme molecules incorporated within each complex by florescence imaging. Although both single enzymes and multiple-enzyme complexes appear as individual diffraction-limited spots, their fluorescence intensities differ depending on the number of labeled enzymes per complex. We thus measure the background-subtracted fluorescence intensity of each particle in the first frame of the image sequence and compare the resulting intensity distributions for single-enzyme particles and multienzyme complexes. For single enzymes, the intensity distribution appears Gaussian with a single peak around 6000 AU in intensity [Figure 4B­(i) and Supp. Table 7]. Interestingly, the distribution for multiple-enzyme complexes retains the same initial peak at 6000 AU and exhibits higher intensity values up to about 8 times the initial peak intensity [Figure 4B­(ii) and Supp. Table 8]. Given the labeling efficiency of urease ranges between 0.1 and 0.7 (i.e., less than one fluorophore per enzyme; see Supp. Methods), we speculate that the single-peaked intensity distribution of single enzymes primarily arises from single urease molecules labeled with one fluorophore, with occasional double labeling accounting for the small tail extending to ∼15000 AU. The width of the distribution likely reflects the intrinsic fluorescence fluctuations of individual fluorophores. In contrast, the wider distribution observed for multiple-enzyme complexes implies the presence of several urease molecules conjugated within each complex. Further quantification of the exact number of enzymes per complex is challenging, as individual enzymes may carry varying numbers of fluorophores (ranging from 0 to 2 or even more). Nevertheless, the emergence of intensity distributions that extend to much higher intensity values confirms the presence of multiple enzymes within individual complexes and validates our assembly strategy.

As before, we perform single-particle tracking experiments to determine the diffusion coefficients of the multiple-enzyme complexes in the absence or presence of 1 mM urea. We observe a statistically significant shift in the distribution of measured diffusion coefficients in the presence of 1 mM urea (Figure 4C). Using the CDF, we determine the median and find an increase of 150% (*p* = 1.2 × 10^–6^, KS Test) comparing 0 to 1 mM urea for the multiple-enzyme complexes (Figure 4D).

We also find that the median diffusion coefficient of the multienzyme complexes is much lower (∼17%) than that of single enzymes tethered via biotin–streptavidin for the same enzyme preparation (compare Figures 4C to 2B). This reduction in mobility is likely due to the larger hydrodynamic radius of the enzyme multimers. Performing an estimate of the expected diffusion coefficient based on the deduced percentages of monomers and each type of multimers, we find an expected reduction to ∼70% of the single-enzyme value. An alternative possibility for the observed reduction in diffusion is that multiple-enzyme complexes may bind to the lipid bilayer through several biotin–streptavidin connections, introducing additional drag from the lipids.^35^ This scenario is illustrated in Figure 4A (leftmost complex), where a multienzyme complex containing several streptavidin molecules anchors to the bilayer at multiple points simultaneously, which could further reduce the basal diffusion coefficient. Overall, our findings suggest that assembling multiple enzymes into larger complexes may provide a viable strategy for engineering nanoscale active particles with controllable enhanced diffusion.

In summary, we present a new set of experiments to test the phenomenon of enzyme-enhanced diffusion by tethering enzymes to a fluid lipid bilayer and characterizing their motion using single-particle tracking. The goal of the tethering is to both restrict the motion to two dimensions and to increase the drag to slow dynamics and increase the period of observation. It also allows for complementary tethering schemes. Lipid-tethered enzymes exhibit substrate-dependent enhanced diffusion—an increase in diffusion rate driven by catalytic turnover. Using two complementary tethering strategies, we show that the diffusion coefficient of single enzymes increases by ∼40%, independent of the specific conjugation method. This enhancement aligns with previous reports of increased diffusion in free enzymes.^2,5,12–14^ Importantly, the effect scales with substrate concentration and disappears when enzymatic activity is inhibited, further supporting a catalysis-dependent mechanism.

Remarkably, we find that larger particles created by random cross-linking of multiple urease enzymes via biotin–streptavidin exhibit greater enhancement than single enzymes. While this work does not dissect the possible mechanisms behind this enhancement, it points to a practical use for enzymes in powering active motility of objects from the nanoscale to the microscale. Indeed, prior work has already shown that microscale and macroscale objects can be powered by enzymatic reactions.^8,36–39^ Future work could combine these enzymes with other nanoscale building blocks, such as polymers,^40^ nanocolloids,^41^ or DNA origami^21,42^ to create customizable active particles that could be the basis for biocompatible, active materials of the future.

Recent studies employing complementary single-molecule techniques, such as anti-Brownian electrokinetic (ABEL) trap and single-molecule displacement/diffusivity mapping (SMdM), have reported no catalysis-dependent enhancement of enzyme diffusion in freely diffusing systems.^17,20^ In contrast, our current and previous results consistently show a 30% to 3-fold increase in enzyme diffusivity upon substrate catalysis when enzymes are either slowed by viscous agents (e.g., methylcellulose) or tethered to fluid lipid bilayers.^4^ In both cases, the baseline diffusivity (*D*_0_) of the enzymes is substantially reduced. We therefore hypothesize that the apparent discrepancy between our findings and earlier observations arises from differences in the baseline thermal motion of the system. As the origin of “enhanced diffusion” likely involves transient bursts of local stress, conformational changes, or electrostatic perturbations transmitted to the surrounding fluid—mechanisms that remain under debate.^13,43^ We speculate that viscous environments or lipid tethering may prolong these transient couplings to allow the active component of motion to make a larger contribution to the mobility compared to the background thermal fluctuations, leading to a discernible enhancement in diffusivity. Specifically, when the baseline enzyme diffusion is relatively high, such as when an enzyme is freely diffusion in water, any weak active contribution may be masked by Brownian motion and thus remain experimentally undetectable. In contrast, when the baseline mobility is reduced, such as in viscous or membrane-tethered environments, even modest active effects could become more pronounced and experimentally resolvable. This interpretation suggests that the magnitude of catalysis-dependent diffusion enhancement may depend not only on the enzymatic activity but also on the environmental context in which the enzyme operates. Taken together, our findings, along with previous reports,^17,20^ suggest that crowded or viscous environments that reduce baseline enzyme mobility are critical for observing enhanced diffusion—an interesting result given that many natural and cellular environments are inherently crowded.

## Supplementary Material



## Notes

Detailed experimental methods, additional data figures and tables of the best-fit parameters, and statistical analysis for all data presented (PDF)
