# Supplementary material for: Enhanced Diffusion of Single, Lipid-Tethered Enzymes
Source: Nano Lett. 2026 Feb 14;26(7):2458–65. doi: 10.1021/acs.nanolett.5c05619 (PMC12947734; doi:10.1021/acs.nanolett.5c05619)
Supplement: Supplementary file 1 [file nl5c05619_si_001.pdf]

Supplemental Information for Enhanced Diffusion of Single, Lipid-Tethered Enzymes

Ashley Scott,[†,§] Mengqi Xu,[†,§] Ian Murphy,[‡] David Jang,[†] Zainab Marwa Rana,[†] Anthony Estrada,[†] Wylie Ahmed,[¶] W. Benjamin Rogers,[‡] and Jennifer L. Ross[*,†]

†*Physics Department, Syracuse University, Syracuse, New York, 13244 USA*

‡*Martin A. Fisher School of Physics, Brandeis University, Waltham, MA 02453 USA*

¶*Laboratoire de Physique Théorique and MCD, Centre de Biologie Intégrative, Université de Toulouse, CNRS, UPS, Toulouse, France, and Department of Physics, California State University Fullerton, CA 92831 USA*

§*These authors contributed equally to this work*

E-mail: jlross@syr.edu

Supplemental Information

Detailed Methods

Supported Lipid Bilayers We make lipid bilayers by fusing small unilamellar vesicles (SUVs) made of 1-palmitoyl-2-oleoyl-glycero-3-phosphocholine (POPC) mixed with 0.5 mol% biotinylated- 1,2-dioleoyl-sn-glycero-3-phosphoethanolamine-N-(cap biotinyl) (Biotin-PE) to the experimental chamber surface.[1] All of the lipids are purchased from Avanti Polar Lipids. These lipids are thoroughly mixed in a glass test tube, resulting in a total volume of 107.5 μl. Next, the mixed solution is evaporated under N_2 gas, leaving a thin film at the bottom of the tube. The dehydrated solution is placed in a vacuum desiccator for 4 hours to remove

any remaining choloroform. The completely dried lipid is then hydrated with 500 μl of 1X TE buffer (10 mM Tris base and 1 mM EDTA, pH 8 with HCl), and this solution is left to incubate overnight at room temperature. When hydrated, the lipid appears cloudy due to the formation of Giant Unilamellar Vesicles (GUVs). The following day, the lipid solution is sonicated using a tip sonicator for 2 minutes until the solution appears clear due to the GUVs breaking up into SUVs. We then centrifuge the lipid solution for 10 minutes at 5000 rcf; the centrifugation process creates a pellet of any impurities remaining in the lipid solution. The supernatant is removed and placed in another tube, and the centrifugation process is repeated 3 times. After the final centrifugation step is complete we add an additional 500 μl of 1X TE buffer to bring the final concentration to 1.03 mg/ml.

Chamber preparation Chambers are made from a slide and coverslip, washed with ddH_2O water, then washed with ethanol, and then washed with acetone. The slides are dried using air current of dry N_2 gas and then placed into a UV-Ozone machine (Jelight) for 20 minutes. Immediately after the UV-Ozone treatment, the slide and coverslip are removed and used to create a flow chamber with two pieces of permanent double-stick tape (3M) creating a path through which fluids can be flowed.

Biotin-Streptavidin Tethering To tether enzymes to the surface and visualize them in single molecule imaging, we biotinylate urease from jack bean using a commercially sourced EZ-Link Sulfo-NHS-LC-Biotinylation kit (ThermoFisher, Cat 21435), which uses succinimidyl esters to label surface lysines and the amino-terminus, following the instructions provided. Briefly, 2 mg/ml fluorescently labeled urease was mixed with 20-fold molar excess Sulfo-NHS-LC-Biotin solution, incubated at 4°C overnight, and purified with Zeba Spin Desalting Column (Thermo Scientific). The protein was stored at 4°C, protected from light, and used within three days to ensure stability and activity.

We fluorescently label the urease with two different approaches. One approach is to label surface cysteine groups through the maleimide linker (ThermoFisher, Cat A20347) and another is to label surface lysines via succinimidyl esters (ThermoFisher Cat A20173). For

each, we used the commercially available protein labeling kit (ThermoFisher Cat A20173) following the optimized protocols provided. Briefly, 2-5 mg urease powder was dissolved in 1 mL of 1× PBS buffer (0.137 M NaCl, 0.0027 M KCl, and 0.0119 M phosphates, pH 7.4, 10× PBS buffer was purchased from Thermo-Fisher), and 100 μL of 1 M bicarbonate (pH 8.3) was added to raise the pH to 7.5–8.5, optimizing reactivity for maleimide or succinimidyl esters. The enzyme solution was transferred to a vial containing Alexa Fluor 647 reactive dye (urease to dye molar ratio = 1:3), mixed thoroughly, incubated at room temperature for 2 hr, and purified with Bio-Rad BioGel P-30 Fine size exclusion purification resin (provided by ThermoFisher in the protein labeling kit Cat A20173). The degree of labeling was determined by measuring the absorbance at 280 nm and 650 nm. The ratio of label per urease varies from 0.1 to 0.7 depending on the dye used and if the enzyme was previously labeled with biotin or DNA. Urease was stored at 4°C, protected from light, and used within three days after preparation. Note, when we used the succinimidyl ester for both the Alexa and the biotin labeling, the efficiency was very low. Thus, for biotin-streptavidin experiments, we used the maleimide dye for the Alexa labeling and the NHS (succinimidyl ester) biotin kit for the biotin labeling to achieve a labeling efficiency of 0.1-0.7 per urease hexamer.

The linker between the biotinylated lipid and the biotinylated enzyme is streptavidin, which we add into the chamber to bind to the biotin on the membrane and wash with seven chamber volumes. Finally, we add the biotinylated enzymes and wash with seven chamber volumes.

Cholesterol-DNA Tethering For this approach, we use a different formulation of the lipid bilayer.[2] Specifically, the lipid mixture is composed of 97.1% (w/w) 1,2-dioleoyl-sn-glycero-3-phosphocholine (18 : 1 DOPC, Avanti Polar Lipids), 2.4% (w/w) 1,2-dioleoyl-sn-glycero-3-phosphoethanolamine-N-[methoxy(polyethylene glycol)-2000] (18:1 PEG2000 PE, Avanti Polar Lipids), and 0.5% (w/w) Texas Red 1,2-dihexadecanoyl-sn-glycero-3- phosphoethanolamine (Texas Red DHPE, Thermo Fisher Scientific). The PEG on the lipid is used to keep the DNA from sticking directly to the lipid, as previously shown.[2] We anchor

the DNA receptors using a cholesterol anchor formed from a cholesterol-triethylene glycol (TEG)-modified single-stranded DNA molecule (Integrated DNA Technologies, Inc.), and will henceforth refer to these anchors as the cholesterol-DNA. The urease-bound complementary DNA strand can bind to the cholesterol-DNA via DNA hybridization (main text Fig. 1B). As above, the supported lipid bilayer is created by adhering SUVs to a glass surface, and the lipid vesicles are created using the evaporation method described above. The final lipid solution is prepared by mixing 100 μl of 18:1 (Δ9-Cis) PC DOPC at [10 mg/mL], 5 μl of DHPE Texas Red lipids at [1 mg/mL], and 2.5 μl of 18:1 PEG2000 lipids at [10 mg/mL].

We purchased two ssDNA oligonucleotides from IDT: a 78-base pair sequence (5'-/TTT CAA CCA TCA CGA ATA CAT TTT TTT TTT TTT TTT TTT TTT TTT TTT TTT TGT CCT TTT AGG AAG AGA ATG GTT - 3') modified with Alexa 647 at the 3' end, and a second 17-base pair sequence (5'-/TTT TGT ATT CGT GAT GGT TGT TT/- 3') modified with 6-FAM at the 5' end and a cholesterol-TEG modification at the 3' end. The ssDNA is purchased as a 100 μM solution in 1X TE buffer at pH 8 (IDT). For our experiments, we prepare a 20 μM stock solution by annealing the DNA strands in a PCR program, starting at 95°C and gradually decreasing to 25°C over a period of 1 hour and 30 minutes. For use in the experiments, the 20 μM stock solution is then diluted to 2.5 μM.

As described in our recent paper,[3] we use a commercially available heterobifunctional crosslinker, azido-PEG3-maleimide (Vector Laboratories, CCT-AZ107), to connect synthetic single-stranded DNA to surface-exposed cysteine groups on the urease. The maleimide functional group on the crosslinker reacts with the cysteine groups, forming a thiosuccinimide linkage. We introduce single-stranded DNA modified with a dibenzocyclooctyne (DBCO) molecule (DBCO-DNA) to undergo a strain-promoted click reaction with the azide functional group on the other end of the crosslinker, resulting in a stable covalent bond.[4]

Chamber preparation for biotin-stpretavidin linkers To make supported lipid bilayer flow chambers, 10 μl of SUV solution is first flowed in and incubated for 20 min to

allow for the fusion of SUVs to the surface. Excess unfused SUVs are subsequently removed by washing the chamber with 50 μl 1X PBS buffer. The lipid bilayer-coated chambers are kept in a humid container to prevent dehydration and taken out immediately before use.

To image single biotinylated urease enzymes, we loaded 0.01 mg/ml streptavidin into the biotinylated lipid coated chamber first, incubated for 5 min. Excess unbound streptavidin is subsequently removed by washing the chamber with 50 μl 1X PBS buffer. Next, 14 μl of 100 pM Alexa647-biotin-urease (diluted in 1X PBS buffer) is flowed in the chamber and incubated for 5 min to allow for binding. The free unbound Alexa647-biotin-urease is washed by 50 μl 1X PBS buffer. To create multiple enzyme complexes, we pre-incubated biotinylated urease with streptavidin and loaded the urease-streptavidin mixture into the biotinylated lipid coated chamber. Specifically, 5.38 μM Alexa647-biotin-urease is mixed with a 2-fold molar excess of streptavidin (SA) and incubated on ice for 1 hr to form SA-Alexa647-biotin-urease complexes. The complex mixture is then diluted by 10,000 times to make the enzyme concentration optimized for single particle imaging. The diluted complex mixture was then flowed into the biotinylated lipid bilayer-coated chamber and incubated in a humid container for 10 min. Free unattached streptavidin and enzyme complexes are then removed by washing with 50 μl 1X PBS.

Right before imaging, the reaction mixture containing 1 mM urea, 10 mM dithiothreitol, 15 mg/ml glucose, 0.15 mg/ml catalase, and 0.05 mg/ml glucose oxidase in 1X PBS, is loaded into the chamber and sealed with epoxy. For all chambers, an oxygen scavenging system with 10 mM dithiothreitol (DTT), 15 mg/ml glucose, 0.15 mg/ml catalase, and 0.05 mg/ml glucose oxidase is added to extend the lifetime of the fluorescent dyes and minimize photobleaching.

Chamber preparation for cholesterol-DNA linkers To prepare supported lipid bilayer flow chambers, we dilute the 1.03 mg/ml working stock solution of SUVs with PEG to 0.75 mg/ml. Then, 13 μl of the diluted solution is flowed into the chamber, and the sample is incubated for 30 minutes to allow the lipid bilayer to form on the chamber surface. During

incubation, the sample is kept in a humidified container to prevent dehydration. Afterward, the sample is washed with 130 μl of 1X PBS (with adjusted pH 6.7) buffer to remove any unbound SUVs. Note the PBS used for DNA-systems is lower in order to prevent DNA unbinding. Next, hybridized DNA receptors are added to the chamber at a concentration of 2.5 μM and incubated for 10 minutes to allow binding to the lipid bilayer. The sample is then washed three times with a total of 39 μl of 1X PBS buffer to remove any excess unbound receptors. Finally, labeled urease is added to the chamber at a final concentration of 50 pM.

Plotting and Fitting FRAP Data Fluorescence recovery after photobleaching (FRAP) of the lipid was performed daily to get a baseline for the diffusion coefficient and would be performed again, if needed. The FRAP intensity data was quantified over time using FIJI/ImageJ by outlining a circle over the region of interest (ROI) of the photobleached area and measuring the area and intensity of the ROI over the entire movie (Supp Fig. 1A).

During data collection, there is global photobleaching of the fluorophores over time, which is obvious when the raw, original intensity data is plotted vs. time (Fig. 1Bi). To correct the global photobleaching, we perform a correction analysis. First, the maximum intensity prior to photobleaching, I_{max} is subtracted from the intensity over time, $I(t)$ to give $I_{adjust}(t) = I_{max} - I(t)$. The adjusted intensity values are plotted over time and fit using a rising exponential function: $y = A(1 - B\exp(t/\tau)$, where the fit parameters A and B are typically close to 1. Data points during the photobleach are masked during this process to ensure a better fit to global photobleaching (Fig. 1Bii). Next, the fit equation for the adjusted data is added back to the original intensity data to correct for the global loss of intensity (Fig. 1Biii). We check that the photobleaching correction shows that the data appears to level off before the local photobleaching and after the recovery. The data is then shifted in time and intensity so that the minimum intensity value is situated at the origin (Fig. 1Biv).

In order to directly use the data to determine the recovery rate and percent intensity

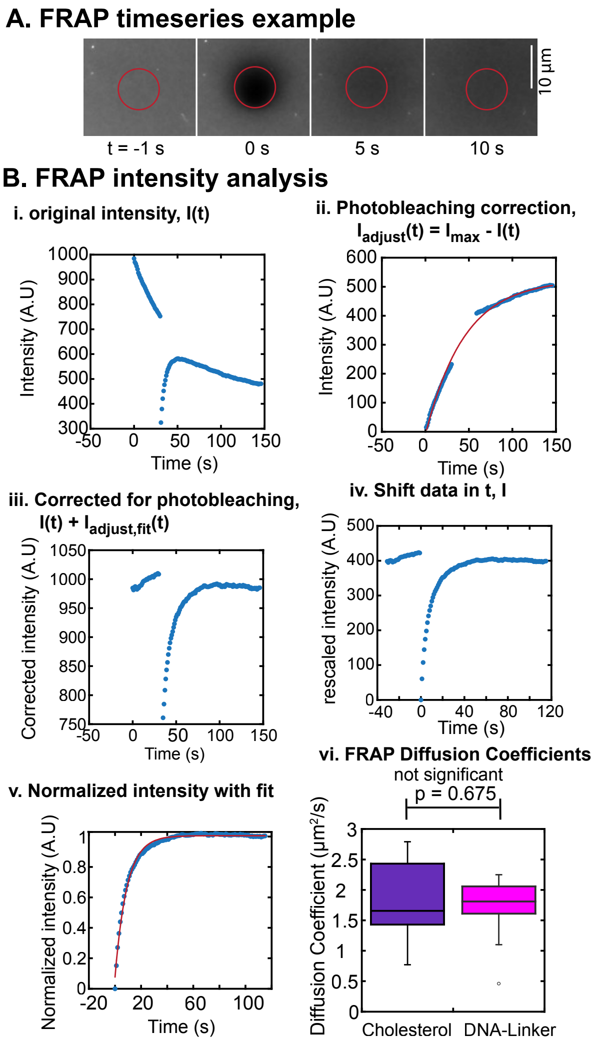

Figure 1: Fluorescence Recovery After Photobleaching Methods. (A) Timeseries of images of fluorescently-labeled cholesterol photobleached in the circular ROI over 10 seconds. Scale bar is 10 μm. (B) FRAP intensity analysis with photobleaching correction. (i) Original fluorescence intensity in ROI ($I(t)$) over 150 s. (ii) Plot of $I_{adjust}(t) = I_{max} - I(t)$ used to fit the data to a decaying exponential to remove the global photobleaching. Best fit parameters are given in Supp. Table 1. (iii) Correcting the intensity to remove the global photobleaching by adding the fit equation from (ii), $I_{adjust,fit}(t)$ to the original intensity data, $I(t)$. (iv) The corrected data is shifted in x and y to set the minimum intensity value to zero at time zero. The location of the minimum intensity value and time from (iii) is subtracted from the data. (v) The data is normalized by dividing all the data by the initial average intensity prior to bleaching. This data is fit to equation 1. Best fit parameters are given in Supp. Table 1. (vi) Box-whisker plot of FRAP results comparing cholesterol and DNA-linkers in the same samples. There is no significant difference.

recovery, it is easiest to normalize the data so that the average initial intensity is equal to 1 by rescaling the shifted data. In the example shown in figure 1, the data is divided by about 400 to bring the initial intensity average to one. We can see that the final recovery is also one, showing that the recovery was complete within the time we observed.

To determine the recovery rate and the percentage of recovery, the normalized intensity data is plotted over time and fit to a recovery equation the form:

$$y(t) = A(1 - B(exp(-t/\tau))) \tag{1}$$

where A is the amplitude, B is a coefficient that scales the initial offset after photo-bleaching and τ is the characteristic time constant. The time constant, τ is used to determine the two-dimensional diffusion coefficient as so: $D = r^2/4\tau$, where r^2 is the radius of the ROI used for the analysis measured in microns.[2,5,6]

FRAP data was taken for fluorescent lipids with and without 1 mM urea to ensure that the urea is not affecting the mobility of the lipids (Supp. Fig. 4). We also perform FRAP with a high concentration of cholesterol and DNA linkers (Main text Fig. 1D).

Single Molecule Imaging To visualize the motion of enzymes, we employ single-molecule imaging using total internal reflection fluorescence microscopy (TIRF). In this technique, a laser is directed at the glass-water interface at an oblique angle, creating an evanescent wave of light that penetrates only about 200-400 nm from the surface. Fluorescent molecules within this range are illuminated. The TIRF system used consists of an inverted Nikon Eclipse Ti2 microscope equipped with an iLas (GATACA Systems) attachment for laser illumination, utilizing the ring TIRF modality to ensure a smooth illumination region. Enzymes are excited with a 65 mW, 640 nm red laser to excite the AZDye 647 fluorophores, and the diffusion of these particles are recorded with a Prime BSI camera (Teledyne Photometrics). Movies are captured over 30 seconds at a frame rate of 60 ms.

Particle Tracking Prior to tracking, each single particle is cropped to contain the entire

trajectory in the cropped frame and the particle image is enhanced using a 2D Gaussian filter using the SpotTracker plugin in FIJI with setting the diameter to be 5-6 pixels.[7]

Single-particle tracking is performed using the Particle Tracker 2D/3D plugin from the Mosaic Tool Suite in FIJI.[8] The parameters for this plugin are divided into two categories: Particle Detection, which includes Radius, Cutoff, and Percentile; and Particle Linking, which includes Displacement and Link Range. Radius refers to the approximate size of the particle in pixels, determined from the diffraction-limited imaging. Cutoff defines a threshold for discriminating non-particles, specifically if an object is larger than this cutoff, then it is excluded. Percentile determines which bright spots are considered particles by setting a threshold on the minimum intensity of the particle being tracked. In Particle Linking, which describes the ability to track a particle from one frame to another, the Link Range specifies how many future frames are expected in the trajectory. Displacement sets the maximum number of pixels a particle is allowed to move between two consecutive frames. For our data, the tracking parameters are set as follows: Radius is set to 5-6 pixels, Cutoff is set to 3, Percentile varies between 0.01-0.1, and both Linking Range and Displacement are set to 6-7 frames. The plugin also includes a feature to allow for the researcher to check the accuracy of the particle detection frame-by-frame before using the track to ensure the particle is being tracked well for the entire length of the video. We check this for each particle prior to including in the data set. Data is saved as text files (.csv) and saved individually for mean-squared displacement analysis.

Mean Squared Displacement The trajectories determined from particle tracking are used to calculate the time-averaged mean squared displacement (MSD) using MATLAB, as previously described (Fig. 2Bi).[9] During the tracking, the particle will photobleach, which decreases the localization efficiency. Additionally, the error in the MSD increases with lag time as there are fewer points to average over. We had the code plot and fit to a line: $MSD = 4D\tau$, where D is the diffusion coefficient and τ is the lag time (Fig. 2Bii). In order to automatically find the best fits, we fit the line equation to the data with an increasing

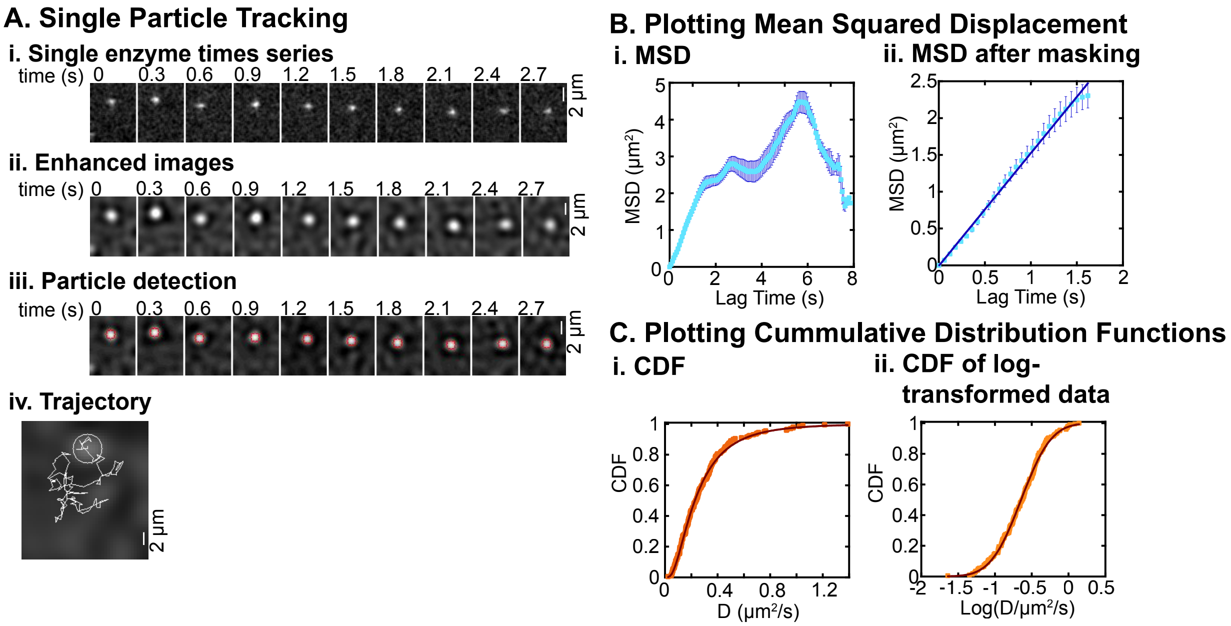

Figure 2: Single particle tracking and mean squared displacement method details. (A) Single particle tracking (i) Example timeseries of a particle tracked over 2.7 s. (ii) Images are enhanced using the SpotTracker 2D Gaussian filter. (iii) Particle Tracker is used to find the center of each particle with sub-pixel precision. (iv) Complete trajectory of particle over over 100 frames. All images have scale bar of 2 μm, as shown. (B) Mean squared diffusion example of single trajectory. (i) MSD of the particle trajectory shown in (A) over 8 seconds. (ii) Data from Bi masked so that only first 28 points (1.5 s) are shown and fit to a line to find D. Best fit given in Supp. Table 11. (C) At least 100 molecules are tracked and diffusion coefficients determined to compare experimental parameters. (i) CDF of untransformed data shows a log-normal distribution. (ii) Log-transformed data CDF appears normally distributed.

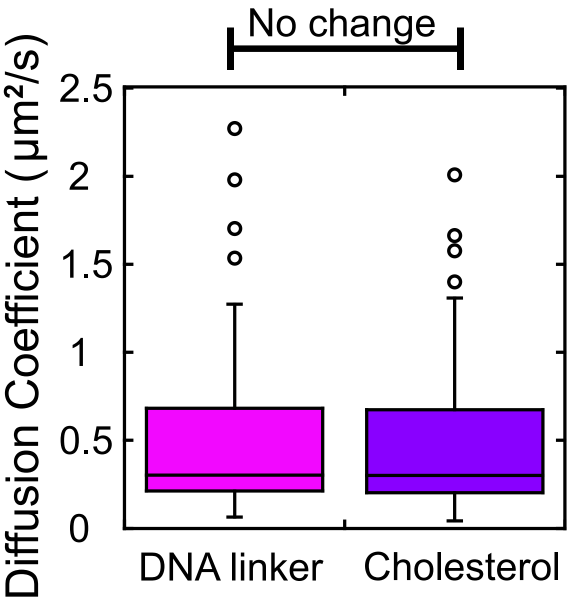

Figure 3: Box-whisker plots of diffusion data for single particle complexes of cholesterol (purple) and DNA (magenta).

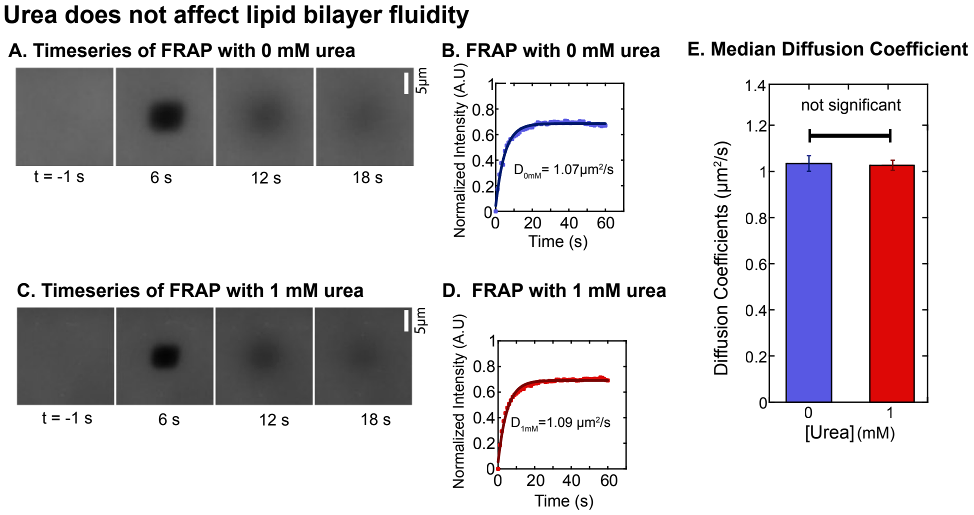

Figure 4: Urea has no effect on lipid bilayer fluidity. (A) Time series of photobleaching and recovery of the lipid bilayer with 0 urea. (B) Fluorescence intensity recovery over time of 0 mM (blue squares) fit to exponential rise (black line). Fit parameters are given in Supp. Table 13. (C) Time series of photobleaching and recovery of the lipid bilayer with 1 mM urea. (D) Fluorescence intensity recovery over time of 1mM urea (red squares) fit to an exponential rise (black line). Fit parameters are given in Supp. Table 13. (E) Bar chart of median values comparing 0 mM urea (blue bar) and 1 mM urea (red bar) of 9 average FRAP experiments. These data are statistically indistinguishable, as the probability that they are the same is 99% using the KS-Test.

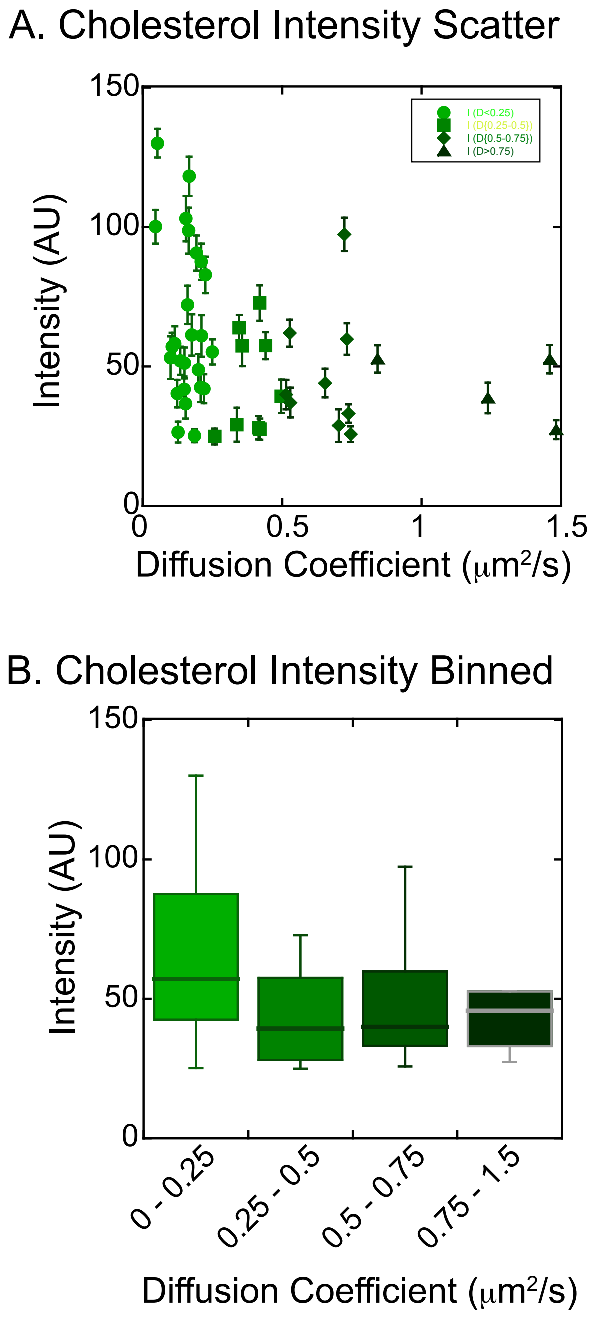

Figure 5: Single particle tracking and intensity measurement of labeled cholesterol. (A) Single particle tracking was used to determine the diffusion coefficient and intensity line scans fit to Gaussian functions were used to determine the intensity of each cholesterol particle tracked. The intensity of each spot was plotted as a function of the diffusion coefficient. The slower diffusing particles have higher intensity and the fastest molecules have lower intensity on average. (B) Box-whisker plots of the intensity grouped over ranges of diffusion coefficients show that the slowest cholesterol particles have increased intensities. Note that because there are only 4 molecules in the last box, the upper error bar and the top of the box are the same number.

number of data points used in the fit ($N = 7$ or greater). We use a cutoff on the goodness of fit where data was required to have $R^2 \geq 0.94$ to be included. Further, the fit data must have at least 7 or more data points (at least 0.42 s of data) to be included.

Intensity Measurements The intensity of biotin-streptavidin enzyme complexes are measured using FIJI/ImageJ. Single particles are identified in the first frame of each movie by binarizing the image, generating a mask from the binary output, and applying the "Analyze Particles" tool in ImageJ to detect individual regions of interest (ROIs). Then, total fluorescence intensity within each particle region is measured, and a corresponding background intensity is determined by shifting each ROI to a nearby region of the same size and calculating the mean intensity in that region. The background-corrected intensity is then calculated using the following formula: $I_{corrected} = I_{total} - area * I_{background,mean}$, where $area$ is the area of the particle region.

The obtained intensity distributions were fitted to a single Gaussian (Eqn. 2):

$$y = B + A\exp(-(x-\mu)^2/(2*\sigma^2)) \tag{2}$$

where B is the background intensity, A is the amplitude, μ is the mean value, and σ is the standard deviation. For the fitting of the intensity data, B was set to zero. For the multiple enzyme dataset, we fixed the mean value and standard deviation but allowed for the amplitude to vary. The best fits are given in tables 7 and 8.

The intensity measurements of cholesterol particles was determined via a second method where the intensity profile of the particle image is created using the plot profile function in ImageJ. The intensity profile is fit to a Gaussian function of equation 2 allowing B to be a fit parameter. The amplitude is used as a measure of the intensity and compared between different data sets. In this method, the background is already removed because the fit estimates it separately.

Enzyme Inhibition We mix 20 μl of 12 μM DNA-urease conjugate with 100 μl of

HEPES buffer (20 mM HEPES, pH 7.5), yielding a final urease concentration of 2 μM. The inhibitor, catechol (Fisher Cat P056725G) is a powder that is hydrated in DMSO to 500 mM stock. A 16 μM catechol solution is prepared by combining 3.84 μl of the stock solution of catechol with 116.16 μl of HEPES buffer. We then mix 120 μl of catechol solution with 120 μl of the urease solution, resulting in a final mixture of 8 mM catechol (8000 catechol molecules per urease hexamer). The reaction mixture is incubated in the dark for 30 minutes. Following incubation, a 100 kDa MWCO filter is pre-washed with phosphate buffer (10 mM Na_2HPO_4, 1.8 mM KH_2PO_4, 137 mM NaCl, 2.7 mM KCl, pH 6.7). The urease-catechol mixture (240 μl total volume) is then washed three times at 9,400 x g for 8 minutes each, adding fresh phosphate buffer after each spin. After the final wash, the urease solution is re-diluted with 175 μl of phosphate buffer and stored at 4°C until use.

Enzyme Activity Assays We prepare enzyme samples at a final concentration of 28 μM phenol red and 5 mM urea in a phosphate buffer at pH 6.7. We add to these solutions DNA-modified urease, either active or inhibited, to a final concentration of 5 nM. For reference, we include blanks with the same phenol red and urease concentrations without urea. Immediately after the addition of urease, we seal the plate with a transparent plate sealer (Thermo Scientific 1424518), and we move the plate to an EPOCH2 microplate reader (BioTek) that takes absorbance measurements of each sample at 560 nm every 60 seconds for up to 90 minutes at 28°C.

Data Tables

Additional information on data shown in the main document are given here including the fit equations and number of points for data in all figures.

Table 1

Best fits for data in Figure 1Cii and Diii the fit equation for fitting FRAP data, equation 1

data	fit	A	B	τ	χ^2	R^2
Fig. 1Cii	Eq. 1	0.8804 ± 0.0002	0.87 ± 0.01	10.1 ± 0.3	0.05	0.990
Fig. 1Diii, chol	Eq. 1	0.977 ± 0.002	0.92 ± 0.01	9.3 ± 0.2	0.02	0.996
Fig. 1Diii, DNA	Eq. 1	0.981 ± 0.002	0.96 ± 0.01	6.5 ± 0.1	0.03	0.994

Table 2

Best fits for data in Figure 2Aiii using the equation for a line: y = mx + b

data	Slope(m)	Intercept	χ^2	R^2
0 mM	1.44 ± 0.01	-0.14 ± 0.02	0.07	0.998
1 mM	2.52 ± 0.03	-0.16 ± 0.03	0.15	0.998

Table 3

Statistical data for CDF plots in Figure 2Bii.

data	mean	median	standard deviation	standard error of the mean	N
0 mM	1.07	0.526	1.66	0.06	663
1mM	1.37	0.801	1.77	0.06	877

Table 4

Statistical data for CDF plots in Figure 2Cii.

data	mean	median	standard deviation	standard error of the mean	N
0 mM	0.210	0.147	0.202	0.021	91
1 mM	0.264	0.225	0.204	0.020	100

Table 5

Statistical data for CDF plots in Figure 2Bi.

data	mean	median	Rescaled D	standard deviation	SEM	N	P
0 mM	0.374	0.340	0	0.169	0.017	98	1
0.012 mM	0.451	0.398	0.093	0.318	0.034	86	0.322
0.025 mM	0.304	0.261	-0.310	0.184	0.018	98	0.007
0.050 mM	0.403	0.261	0.079	0.189	0.019	99	0.318
0.075 mM	0.513	0.447	0.234	0.296	0.029	100	0.002
0 mM	0.296	0.250	0	0.181	0.018	100	1
0.1 mM	0.312	0.300	0.066	0.157	0.015	100	0.337
0.2 mM	0.368	0.309	0.250	0.211	0.021	100	0.0134
0 mM	0.283	0.240	0	0.182	0.018	100	1
0.5 mM	0.483	0.416	0.662	0.257	0.025	100	0.05e-7
1 mM	0.335	0.319	0.294	0.136	0.013	100	0.07e-9
10 mM	0.325	0.282	0.338	0.168	0.016	100	0.0011
0 mM	0.210	0.146	0	0.202	0.021	91	1
1 mM	0.264	0.226	0.283	0.204	0.020	100	0.00147
100 mM	0.258	0.206	0.291	0.194	0.020	87	0.00609

Table 6

Statistical data for CDF plots in Figure 3Bii.

data	mean	median	standard deviation	standard error of the mean	N
0 mM	0.558	0.480	0.382	0.046	96
1 mM	0.584	0.515	0.375	0.039	92

Table 7

Best fits for data in Figure 4Bi using the equation for a Gaussian using equation 2.

data	A	μ	σ	χ^2	R^2	N
single enzymes	0.47 ± 0.01	5960 ± 40	1620 ± 80	0.002	0.991	247

Table 8

Best fits for data in Figure 4Bii using the single Gaussian with the same mean and standard deviation as Figure 4Bi, but allowing the amplitude to vary.

data	A	μ	σ
monomer	0.23 ± 0.02	5959.2 (fixed value)	1620.5 (fixed value)

Table 9

Statistical data for CDF plots in Figure 4C.

data	mean	median	standard deviation	standard error of the mean	N
0 mM urea	0.355	0.08	0.727	0.05	178
1 mM urea	0.482	0.215	0.676	0.03	484

Table 10

Best fits for data in Supplemental Figure 1Bii and 1Bv the fit equation for fitting FRAP data, equation 1

data	fit	A	B	τ	χ^2	R^2	Area
Fig. 1Bii,chol	Eq. 1	530 ± 2.29	1.01 ± 0.004	47.6 ± 0.7	4594	0.999	56.6
Fig. 1Bv,chol	Eq. 1	1.00 ± 0.002	0.92 ± 0.01	9.1 ± 0.14	0.02	0.996	56.6

Table 11

Best fits for data in Supplement Figure 2Bii using the equation for a line: y = mx + b

data	Slope(m)	Intercept	χ^2	R^2
0 mM urea	1.53 ± 0.02	-0.01 ± 0.02	0.07	0.997

Table 12

Statistical data for diffusion data from Supplemental Figure 3.

data	mean	median	standard deviation	standard error of the mean	N
DNA	0.493	0.302	0.439	0.046	91
cholesterol	0.479	0.300	0.401	0.024	91

Table 13

Best fits for data in Supp. Figure 4 the fit equation for fitting FRAP data, equation 1

data	fit	A	B	τ	χ^2	R^2
Supp.Fig.4Ai, 0mM.	Eq. 1	0.685 ± 0.002	0.937 ± 0.018	4.97 ± 0.164	0.016	0.992
Supp.Fig.4Aiv, 1mM.	Eq. 1	0.691 ± 0.002	0.922 ± 0.02	4.95 ± 0.152	0.013	0.993

Table 14

Statistical data for diffusion data from Supplemental Figure 5B.

data set	mean intensity (AU)	median intensity (AU)	standard deviation	standard error of the mean	N
D < 0.25 $\mu m^2/s$	65.5	57.2	28.5	5.7	25
0.25 < D < 0.5 $\mu m^2/s$	44.5	39.3	18.4	6.1	9
0.5 < D < 0.75 $\mu m^2/s$	47.6	39.9	22.5	7.5	9
D > 0.75 $\mu m^2/s$	42.9	45.7	12.3	6.1	4

References

(1) Kudalkar, E. M.; Deng, Y.; Davis, T. N.; Asbury, C. L. Coverslip Cleaning and Functionalization for Total Internal Reflection Fluorescence Microscopy. *Cold Spring Harbor Protoc.* **2016**, pdb.prot085548.

(2) Merminod, S.; Edison, J. R.; Fang, H.; Hagan, M. F.; Rogers, W. B. Avidity and surface mobility in multivalent ligand–receptor binding. *Nanoscale* **2021**, *13*, 12602–12612.

(3) Murphy, I.; Bobilev, K.; Hayakawa, D.; Ikonen, E.; Videbæk, T. E.; Dalal, S.; Ahmed, W. W.; Ross, J. L.; Rogers, W. B. A method for site-specifically tethering the enzyme urease to DNA origami with sustained activity. *PloS one* **2025**, *20*, e0319790.

(4) Eeftens, J. M.; van der Torre, J.; Burnham, D. R.; Dekker, C. Copper-free click chemistry for attachment of biomolecules in magnetic tweezers. *BMC biophysics* **2015**, *8*, 1–7.

(5) Yoshina-Ishii, C.; Chan, Y.-H. M.; Johnson, J. M.; Kung, L. A.; Lenz, P.; Boxer, S. G. Diffusive dynamics of vesicles tethered to a fluid supported bilayer by single-particle tracking. *Langmuir* **2006**, *22*, 5682–5689.

(6) Kang, M.; Day, C. A.; Kenworthy, A. K.; DiBenedetto, E. Simplified equation to extract diffusion coefficients from confocal FRAP data. *Traffic* **2012**, *13*, 1589–1600.

(7) Sage, D.; Neumann, F. R.; Hediger, F.; Gasser, S. M.; Unser, M. Automatic tracking of individual fluorescence particles: application to the study of chromosome dynamics. *IEEE transactions on image processing* **2005**, *14*, 1372–1383.

(8) Sbalzarini, I. F.; Koumoutsakos, P. Feature point tracking and trajectory analysis for video imaging in cell biology. *J. Struct. Biol.* **2005**, *151*, 182–195.

(9) Michalet, X. Mean square displacement analysis of single-particle trajectories with localization error: Brownian motion in an isotropic medium. *Physical Review E—Statistical, Nonlinear, and Soft Matter Physics* **2010**, *82*, 041914.
